# Hirnabszess als Komplikation bei pulmonaler Manifestation einer HHT

**DOI:** 10.1007/s00108-023-01557-3

**Published:** 2023-07-07

**Authors:** May Cathleen Müller, Christina Weiler-Normann, Mathias Meyer, Christoph Schramm, Gustav Buescher

**Affiliations:** 1 Martin Zeitz Centrum für Seltene Erkrankungen, Universitätsklinikum Hamburg-Eppendorf, Hamburg, Deutschland; 2 I. Medizinische Klinik, Universitätsklinikum Hamburg-Eppendorf, Martinistr. 52, 20246 Hamburg, Deutschland; 3 Klinik und Poliklinik für Diagnostische und Interventionelle Radiologie und Nuklearmedizin, Universitätsklinikum Hamburg-Eppendorf, Hamburg, Deutschland

**Keywords:** Hereditäre hämorrhagische Teleangiektasien, Osler-Rendu-Weber-Syndrom, Genetische Gefäßerkrankung, Arteriovenöse Malformation, Seltene Erkrankung, Hereditary hemorrhagic telangiectasia, Osler–Rendu–Weber syndrome, Genetic vascular disorder, Arteriovenous malformation, Rare disease

## Abstract

Bei einer 43-jährigen Patientin mit einem komplikativen Verlauf eines Hirnabszesses konnte im Martin Zeitz Centrum für Seltene Erkrankungen die Diagnose „hereditäre hämorrhagische Teleangiektasie“ (HHT) gestellt werden. Ursächlich für den Hirnabszess zeigten sich HHT-typische pulmonale arteriovenöse Malformationen (AVM). Bei Patient:innen mit kryptogenem Hirnabszess sollte regelhaft ein Screening auf pulmonale AVM und HHT durchgeführt werden. Die vorliegende Kasuistik verdeutlicht den hohen Stellenwert der ausführlichen Anamnese sowie die interdisziplinäre Behandlung bei komplexen Verläufen seltener Erkrankungen.

## Abstract (en)

A 43-year-old female patient with a brain abscess and a complicated clinical course was diagnosed with hereditary haemorrhagic telangiectasia (HHT) at the Martin Zeitz Centre for Rare Diseases in Hamburg, Germany. The brain abscess was caused by pulmonary arteriovenous malformations (AVM), a typical finding in HHT. Patients with cryptogenic brain abscess should be screened for pulmonary AVM and HHT. This case report illustrates the importance of patient history and interdisciplinary exchange in patients with a broad clinical spectrum as well as interdisciplinary treatment in the case of complications of rare diseases.

## Anamnese

**Figure 1:**
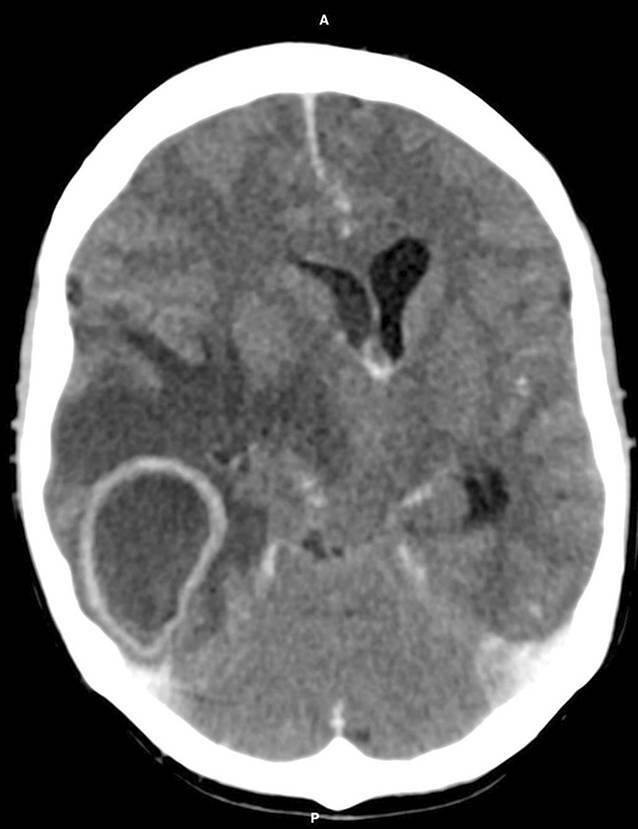

Eine 43-jährige Patientin stellte sich mit starken Kopfschmerzen sowie seit mehreren Tagen anhaltenden Sehstörungen in der Notaufnahme vor. Im kurzfristigen Verlauf entwickelte die Patientin Fieber, eine akute linksseitige Hemiplegie und eine Vigilanzminderung (Glasgow Coma Scale 8). In einer kranialen Notfallcomputertomographie (cCT) mit jodhaltigem Kontrastmittel zeigte sich ein rechtshemisphärischer Hirnabszess mit ausgedehntem, raumforderndem perifokalem Ödem und konsekutiv kritischen Raumverhältnissen (Abb. 1). Im weiteren Verlauf mussten bei zunehmendem Hirndruck eine Hemikraniektomie und Abszessausräumung durchgeführt werden. In den entnommenen Abstrichen konnten mikrobiologisch physiologische Erreger der Mundflora (*Fusobacterium nucleatum, Streptococcus anginosus, Prevotella oralis*) nachgewiesen werden. Anamnestisch war drei Wochen zuvor ein operativer Zahneingriff durchgeführt worden. Nach Konsultation der Mund-Kiefer-Gesichtschirurgie konnte ein ursächlicher oraler Fokus ausgeschlossen werden. Im Anschluss an die intensivmedizinische Therapie wurde die Patientin zur differenzialdiagnostischen Abklärung an das Martin Zeitz Centrum für Seltene Erkrankungen (MZCSE) überwiesen. Zur ambulanten Vorstellung war ein multidisziplinäres Ärzteteam (Innere Medizin, Neurologie, HNO, Humangenetik, Psychosomatik) zugegen. In der Anamnese gab die Patientin an, seit ihrem 14. Lebensjahr an rezidivierender, starker Epistaxis zu leiden. Seit ca. 15 Jahren trete intermittierend Dyspnoe auf. Eine nicht klar zuzuordnende Raumforderung in der Lunge sei vorbekannt und würde regelmäßig radiologisch kontrolliert. Zudem habe sie in letzter Zeit Diarrhöen mit peranalem Blutabgang bemerkt. Familienanamnestisch berichtete die Patientin von der klinischen Diagnose „hereditäre hämorrhagische Teleangiektasie“ (HHT) bei ihrem mit 68 Jahren an einem Apoplex verstorbenen Vater. Auch sei beim Großvater väterlicherseits rezidivierende, schwer stillbare Epistaxis aufgetreten.

## Befund

**Figure 2:**
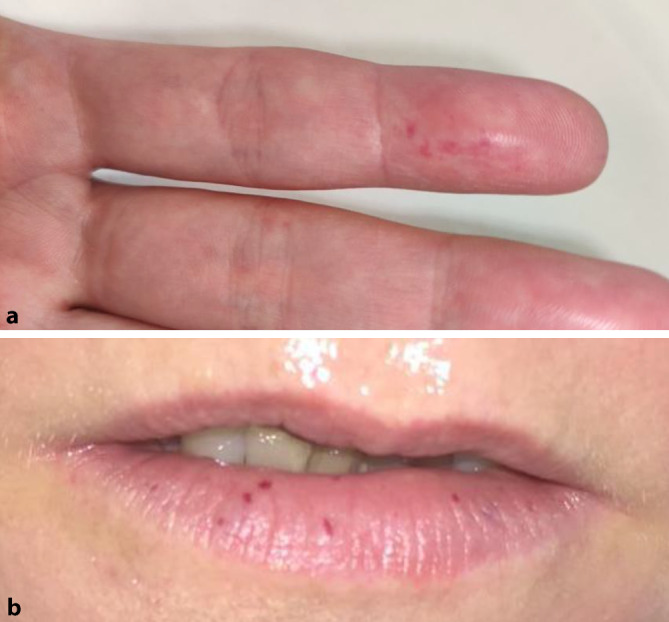

In der körperlichen Untersuchung fielen Teleangiektasien an Wangen, Zunge, Unterlippe und Fingern auf (Abb. 2). In der HNO-ärztlichen Untersuchung zeigten sich Teleangiektasien des Nasopharynx. In der Abdomensonographie wurden leicht dilatierte Lebervenen sowie betonte Periportalfelder festgestellt. In der kontrastmittelunterstützten CT des Thorax konnten drei pulmonale arteriovenöse Malformationen (AVM) identifiziert werden. In der nachfolgenden genetischen Diagnostik mittels Panel-Sequenzierung zeigte sich eine heterozygote Mutation der Variante c.229C>T in *ENG*.

## Diagnose

In Zusammenschau der Anamnese, der klinischen Befunde und der Diagnostik konnte mithilfe der Curaçao-Konsensus-Kriterien bei der Patientin die Diagnose HHT gestellt werden [1].

## Curaçao Consensus Criteria nach Shovlin et al. (2000; [1])

1. Spontane und rezidivierende Epistaxis,
2. mehrere teleangiektatische Veränderungen an (Schleim‑)Häuten an den folgenden charakteristischen Stellen: Lippen, Nasopharynx, Finger, Mundhöhle,
3. eine viszerale Beteiligung in Form von gastrointestinalen Teleangiektasien oder AVM in Lunge, Leber oder ZNS sowie
4. eine erstgradig verwandte Person mit der gesicherten Diagnose HHT

Alle aufgeführten Kriterien erfüllte die Patientin, wobei bei zwei erfüllten Kriterien die Diagnose bereits als wahrscheinlich gilt. Bei mehr als 95 % der HHT-Patient:innen lassen sich Mutationen in den Genen *ENG*, *SMAD4* oder *ACRVL1* feststellen. Die *ENG*-Mutation ist mit der Entstehung pulmonaler und zerebraler AVM assoziiert, während Mutationen in *ACVRL1* häufiger hepatische AVM zur Folge haben. Patient:innen mit *SMAD4*-Mutation zeigen die höchste Rate an Anämien [2].

## Therapie und Verlauf

**Figure 3:**
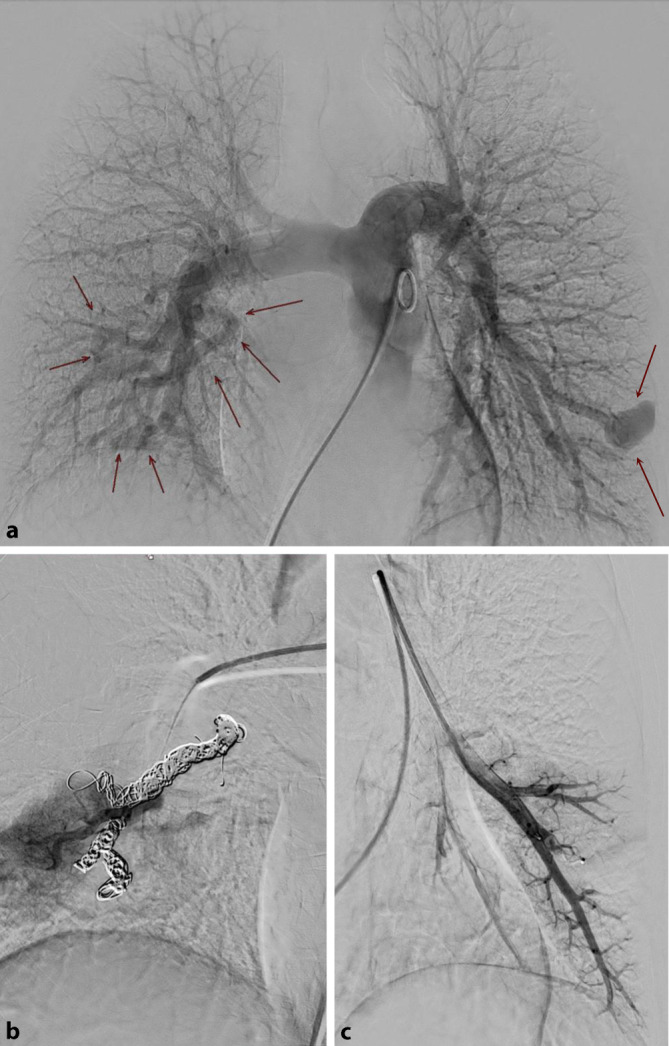

Nach Diagnosestellung erfolgte im MZCSE das weitere interdisziplinäre Management. Die Indikation zur Embolisation der pulmonalen AVM wurde gestellt (Abb. 3a–c). Die HNO-ärztliche Beratung empfahl feuchtigkeitsspendende Nasenpflege sowie die regelmäßige Evaluation der Epistaxis mittels *Epistaxis Severity Score*. Zudem wurden eine Laserbehandlung der nasopharyngealen Teleangiektasien sowie eine medikamentöse *Off-label*-Therapie mit dem VEGF-Antikörper Bevacizumab diskutiert. Die nebenwirkungsarme Therapie mit Bevacizumab zeigte in einer Kohortenstudie hohe Wirksamkeit zur Behandlung schwerer Epistaxis und gastrointestinaler Blutungen [3]. Zur Abklärung gastrointestinaler AVM wurden der Patientin eine zeitnahe Ösophagogastroduodenoskopie sowie Koloskopie empfohlen. Außerdem wurden die konsequente Einnahme einer oralen Eisensubstitution sowie regelmäßige Kontrollen des Eisenhaushalts besprochen.

## Diskussion

Bei der HHT (Osler-Rendu-Weber-Syndrom) handelt es sich um eine autosomal-dominante Erkrankung des Gefäßsystems [2]. Die abnormal formierten Gefäße der Patient:innen befinden sich in (Schleim‑)Häuten, aber auch in inneren Organen wie der Lunge, der Leber oder dem zentralen Nervensystem, wo sie als AVM bezeichnet werden und zu schwerwiegenden Komplikationen führen können [2]. Typische Symptome von HHT-Patient:innen sind rezidivierende, schwer stillbare Epistaxis (90 % der Patient:innen), Teleangiektasien, vorwiegend an Händen und Schleimhäuten, sowie gastrointestinale oder (selten) zerebrale Blutungsereignisse [2, 4]. Auf Basis der suggestiven Krankheits- und Familienanamnese sowie einer körperlichen Untersuchung hätte bei unserer Patientin deutlich früher die Verdachtsdiagnose HHT gestellt werden können. Ursächlich für den Hirnabszess waren im vorgestellten Fall die pulmonalen AVM, die aufgrund der fehlenden kapillären Filterung des Blutes eine Abszessentstehung begünstigen [5, 6]. Aus diesem Grund ist bei HHT-Patient:innen vor allen Eingriffen, die eine Bakteriämie auslösen können, eine Antibiotikaprophylaxe entsprechend der aktuellen Empfehlung zur Prophylaxe der infektiösen Endokarditis indiziert [7]. Die vorgestellte Kasuistik verdeutlicht den hohen Stellenwert der ausführlichen Anamnese sowie den Vorteil einer interdisziplinären Betreuung bei komplexen Verläufen seltener Erkrankungen in einem spezialisierten Zentrum.

Bei Patient:innen mit kryptogenem Hirnabszess sollte ein Screening auf (pulmonale) AVM und HHT durchgeführt werden [8]. Neben dem erhöhten Abszessrisiko prädisponieren pulmonale AVM durch einen erleichterten Übertritt von Emboli in den zerebralen Kreislauf auch für ischämische Schlaganfälle. Aufgrund dieser Risiken tragen pulmonale AVM besonders stark zur Reduktion der Lebenserwartung von HHT-Patient:innen bei und sollten folglich rasch nach Diagnosestellung behandelt werden [9]. Bei frühzeitiger Diagnosestellung, leitliniengerechter Antibiotikaprophylaxe vor dem Zahneingriff und adäquater AVM-Behandlung hätte der komplizierte Verlauf mit hoher Wahrscheinlichkeit vermieden werden können. Der vorliegende Fall stellt ein typisches Dilemma von Patient:innen mit einer seltenen Erkrankung dar. Aufgrund der niedrigen Prävalenz werden oft hochgradig suggestive Befundkonstellationen inadäquat bewertet und eine Diagnose erfolgt erst nach Auftreten von Komplikationen [10].

## Fazit für die Praxis

Bei auffälligen, komplexen Symptomkonstellationen

- ausführliche Krankheits- und Familienanamnese durchführen
- frühzeitigen interdisziplinären Austausch anstreben
- bei Erhärtung des Verdachts auf eine seltene Erkrankung Patient:innen zeitnah an ein spezialisiertes Zentrum überweisen
